# Patient and provider determinants for receipt of three dimensions of respectful maternity care in Kigoma Region, Tanzania-April-July, 2016

**DOI:** 10.1186/s12978-018-0486-7

**Published:** 2018-03-05

**Authors:** M. M. Dynes, E. Twentyman, L. Kelly, G. Maro, A. A. Msuya, S. Dominico, P. Chaote, R. Rusibamayila, F. Serbanescu

**Affiliations:** 1 Centers for Disease Control and Prevention, Division of Reproductive Health, Atlanta, Georgia; 2 Bloomberg Philanthropies Tanzania, Kigoma, Tanzania; 3 AMCA Inter Consult, Dar es Salaam, Tanzania; 4 Thamini Uhai, Kigoma, Tanzania; 5 Kigoma Region Ministry of Health, Kigoma, Tanzania; 6 Ministry of Health Community Development Gender Elderly and Children, Dar es Salaam, Tanzania

**Keywords:** Respectful maternity care (RMC), Disrespect and abuse (D&A), Maternal health, Maternal mortality, Multilevel modeling, Tanzania

## Abstract

**Background:**

Lack of respectful maternity care (RMC) is increasingly recognized as a human rights issue and a key deterrent to women seeking facility-based deliveries. Ensuring facility-based RMC is essential for improving maternal and neonatal health, especially in sub-Saharan African countries where mortality and non-skilled delivery care remain high.

Few studies have attempted to quantitatively identify patient and delivery factors associated with RMC, and none has modeled the influence of provider characteristics on RMC. This study aims to help fill these gaps through collection and analysis of interviews linked between clients and providers, allowing for description of both patient and provider characteristics and their association with receipt of RMC.

**Methods:**

We conducted cross-sectional surveys across 61 facilities in Kigoma Region, Tanzania, from April to July 2016. Measures of RMC were developed using 21-items in a Principal Components Analysis (PCA). We conducted multilevel, mixed effects generalized linear regression analyses on matched data from 249 providers and 935 post-delivery clients. The outcomes of interest included three dimensions of RMC—*Friendliness/Comfort/Attention*; *Information/Consent*; and *Non-abuse/Kindness—*developed from the first three components of PCA. Significance level was set at *p* < 0.05.

**Results:**

Significant client-level determinants for perceived *Friendliness/Comfort/Attention* RMC included age (30–39 versus 15–19 years: Coefficient [Coef] 0.63; 40–49 versus 15–19 years: Coef 0.79) and self-reported complications (reported complications versus did not: Coef − 0.41). Significant provider-level determinants included perception of fair pay (Perceives fair pay versus unfair pay: Coef 0.46), cadre (Nurses/midwives versus Clinicians: Coef − 0.46), and number of deliveries in the last month (11–20 versus < 11 deliveries: Coef − 0.35).

Significant client-level determinants for *Information/Consent* RMC included labor companionship (Companion versus none: Coef 0.37) and religiosity (Attends services at least weekly versus less often: Coef − 0.31). Significant provider-level determinants included perception of fair pay (Perceives fair pay versus unfair: Coef 0.37), weekly work hours (Coef 0.01), and age (30–39 versus 20–29 years: Coef − 0.34; 40–49 versus 20–29 years: Coef − 0.58).

Significant provider-level determinants for *Non-abuse/Kindness* RMC included the predictors of age (age 50+ versus 20–29 years: Coef 0.34) and access to electronic mentoring (Access to two mentoring types versus none: Coef 0.37).

**Conclusions:**

These findings illustrate the value of including both client and provider information in the analysis of RMC. Strategies that address provider-level determinants of RMC (such as equitable pay, work environment, access to mentoring platforms) may improve RMC and subsequently address uptake of facility delivery.

## Plain English summary

Lack of respectful maternity care (RMC) discourages women from seeking facility-based deliveries. RMC is essential for improving maternal and newborn health in sub-Saharan African countries where rates of maternal deaths and non-skilled delivery care are high. We conducted surveys in 61 facilities in Kigoma Region, Tanzania from April to July 2016. Principal components analysis was used to identify three dimensions of RMC. Multilevel regression analyses were conducted on matched data from 249 providers and 935 post-delivery clients. Our outcomes of interest included three dimensions of RMC: 1) *Friendliness, Comfort, and Attention*, 2) *Information and Consent*, and 3) *Non-abuse and Kindness*. Client age, self-reported delivery complications, provider perception of fair pay, cadre, and number of deliveries attended were important factors for receipt of RMC related to *Friendliness, Comfort, and Attention*. Having a birth companion, client religiosity, provider perception of fair pay, and provider age were important factors for receipt of RMC related to *Information/Consent*. Provider age and access to electronic mentoring were important factors for receipt of RMC related to *Non-abusive/Kindness.* Strategies that promote equitable pay, give providers short-term respite away from maternity care, and increase access to mentoring opportunities may improve RMC and uptake of facility delivery.

## Background

Worldwide maternal deaths remain common, with approximately 830 women dying each day from known and largely preventable causes [1]. Access to and use of skilled birth attendance is key to prevention of maternal mortality [2]. Approximately 75–80% [3–7] of maternal deaths worldwide result from obstetric complications and are preventable given access to appropriate interventions. Maternal mortality remains a particularly formidable challenge in Tanzania, where the maternal mortality ratio (556 maternal deaths per 100,000 live births) has demonstrated no detectable reduction over the past 10 years [8]. The percentage of women delivering at a health facility (63%) remains low despite ongoing efforts to increase facility-based delivery [8].

Lack of respectful maternity care (RMC), which includes disrespect and abuse (D&A), has been increasingly recognized [9–14] and demonstrably identified as a key deterrent for women seeking facility-based deliveries [2, 9, 10, 15–28]. Lack of RMC decreases patient satisfaction with services and mediates lack of access to skilled maternity care by reducing the likelihood that patients will return to skilled care for future deliveries [13, 26–28], and by building distrust of facility-based delivery at the community level [29, 30]. Furthermore, lack of RMC may reduce access to appropriate intervention even among patients already within a facility for delivery care by reducing patient-provider communication [31].

The presence of provider-client interpersonal barriers is increasingly suspected to interfere with attempts to increase skilled birth attendance. Bowser and Hill’s review describes seven manifestations of D&A which constitute the current typology in the D&A literature: physical abuse, non-consented care, non-dignified care (including verbal abuse), discrimination, abandonment, and detainment in facilities [11]. Such behaviors are widely recognized to violate patients’ basic human rights. The White Ribbon Alliance (WRA) completed a review of international and multinational human rights instruments related to maternal health rights and the domains of D&A. The resulting Respectful Maternity Care Charter defined seven rights of childbearing women [32] (Table 1): Freedom from harm and ill treatment; Right to information, informed consent and refusal, and respect for choices and preferences, including the right to companionship of choice whenever possible; Confidentiality, privacy; Dignity, respect; Equality, freedom from discrimination, equitable care; Right to timely health care and to the highest attainable level of health; and Liberty, autonomy, self-determination, and freedom from coercion.

**Table 1:** Survey questions and variable names included in the respectful maternity care analyses categorized by White Ribbon Alliance Respectful Maternity Care Charter Article—Kigoma Region, Tanzania, April–July 2016

White Ribbon Alliance Respectful Maternity Care Charter	Survey Question	Corresponding Variable Name
Article I: Every woman has the right to be free from harm and ill treatment	Did any of the health facility staff ever physically abuse you during your visit? By physical abuse, we mean, did they hit, slap, push, kick you, or use any other type of physical force against you.	*Absence of physical abuse*
Article II: Every woman has the right to information, informed consent and refusal, and respect for her choices and preferences, including the right to her choice of companionship during maternity care, whenever possible	Did the staff explain what will happen during your labor and delivery?	*Explain what will happen*
	Did the staff get your consent before proceeding with procedures and exams?	*Consent before procedures/exams*
	Did the staff explain procedures or exams before proceeding?	*Explain procedures/exam beforehand*
	Did the staff inform you of the findings from procedures and exams?	*Inform about findings from procedures/exams*
	Did you feel the information given to you during your visit was too little, just about right, or too much?	*Right amount of information*
	Did the staff ask if you have questions?	*Provider asked if any questions*
	Did the staff encourage you to have a support person with you throughout labor and delivery?	*Provider encouraged companion*
	Did you feel comfortable to ask questions during the visit?	*Client comfortable asking questions*
	Summative Index: Did the staff… • Counsel you about danger signs you should look for in yourself such as too much bleeding, fever, or breast pain? • Counsel you about danger signs you should look for in your baby such as refusing to breastfeed, fever, or convulsions (fits)? • Tell you what to do if you or the baby have any problems? • Counsel you on good body hygiene to prevent infections? • Counsel you on breastfeeding? • Counsel you about exclusive breastfeeding (not using any other fluid/food except breastmilk)? • Ask about your reproductive goals? By reproductive goals, we mean did the provider ask about your desire to have children in the future or to use family planning. • Counsel you on when you can have sex with your husband/partner? • Counsel you on when you can bear another pregnancy? • Counsel you on the risks of sexually transmitted infections (STIs), including HIV? • Counsel you on how to prevent sexually transmitted infections (STIs), including HIV? • Tell you when to return for a follow-up visit?	*Index for receipt of post-delivery counseling*
Article III: Every woman has the right to privacy and confidentiality	Do you believe the information you shared about yourself with the health care provider will be kept confidential?	*Client feels provider will keep information confidential*
	Did the staff provide privacy during counseling or exams such as using a private room, screens, curtains, or cloths to cover you?	*Given privacy for exams or counseling*
	When meeting with the health care provider during the visit, do you think other clients could hear what you said?	*Other clients could not hear discussions*
Article IV: Every woman has the right to be treated with dignity and respect	Did the staff introduce themselves?	*Provider introduced self*
	Did the staff greet you respectfully?	*Greeted respectfully*
	Did any of the staff ever emotionally abuse you during your visit? By emotional abuse, we mean, did they speak or act in an angry or condescending way that made you feel badly about yourself, degraded, embarrassed, or sad?	*Absence of emotional abuse*
	Did the staff interact in a friendly way?	*Interacts in a friendly way*
	Overall, how would you rate the staff’s kindness in the way they spoke to you during this visit?	*Kindness*
	Overall, how would you rate the staff’s level of encouragement during labor and delivery?	*Encouragement*
	How would you rate the facility’s level of cleanliness?	*Level of cleanliness*
	Did the staff advise you on what you could do to make yourself more comfortable when you were in pain?	*Advised of comfort measures*
	Summative Index: What comfort measures did the staff provide to make you more comfortable? • Rubbed back • Offered fluids to drink • Offered food to eat • Assisted in changing position • Helped to walk around • Used encouraging words	*Index for receipt of comfort measures*
Article V: Every woman has the right to equality, freedom from discrimination, and equitable care	None available	None available
Article VI: Every woman has the right to healthcare and to the highest attainable level of health	Did the staff come to attend to you if you called for help?	*Provider came when called*
	Did the staff pay close attention to you throughout labor and delivery?	*Close attention in labor*
	Did the health care staff visit you regularly during the course of labor?	*Visited regularly in labor*
	Was a health care provider with you at the moment of delivery?	*Provider present at delivery*
	Did you feel that your waiting time (when you first arrived at this facility and the time you saw a staff person for a consultation) was reasonable or too long?	*Wait time from arrival to care*
	How long ago was your new baby born? (proxy measure from birth to time from time of exit interview)	*Discharge time 24 h or more after delivery*
	Did the facility provider supplies for your labor and delivery care?	*Facility provided birth supplies*
Article VII: Every woman has the right to liberty, autonomy, self-determination, and freedom from coercion	None available	None available

Worldwide, an alarmingly high prevalence of women have reported mistreatment according to these typologies of D&A, with reports ranging from 20 to 78% [12, 13, 22, 31, 33]. Understanding facilitators of and barriers to RMC is critical to the design of interventions to promote RMC in these contexts.

Qualitative studies have identified several potential patient factors associated with lack of RMC. These include the following: race/ethnicity and religion, depending upon context [34–37]; age, with unmarried adolescents [38, 39] and older women of high parity [40, 41] thought to be at particular risk; socioeconomic status (SES), with poor women at perceived higher risk of D&A [42–45]; and medical conditions, with women with HIV thought to face multiple forms of discrimination [35, 46, 47].

While qualitative studies have identified factors associated with RMC, few studies have quantitatively examined the associations between individual patient characteristics and report of D&A. In Tanzania, women who had attended secondary education or greater, primiparous women, those with experience of low mood in the past year, and those with a personal history of physical abuse or rape were more likely to report experiences of D&A during their delivery; married women were less likely to report D&A [33]. In a follow-up community survey, poor women, women who reported low mood at the time of exit interview, and more educated women were again more likely to report D&A during their delivery, whereas grand multiparas (given birth five or more times) and women with Cesarean sections were less likely to report D&A. Abuya et al., in their exploration of specific forms of D&A during childbirth in Kenya, demonstrated that older women were less likely than younger women to experience non-confidential care, that women of higher parity were more likely to be detained for lack of payment and more likely to have bribes demanded, that married women were less likely to be detained but more likely to be neglected, and that women without a companion were less likely to experience demands for bribes or detention [13].

To our knowledge, published research to date has not modeled relationships between health care provider demographic or practice characteristics and provision of RMC. Qualitative studies, however, including in-depth interviews with providers of maternity care, have generated several hypotheses. Provider training itself is thought to create “distancing” and separation between providers and patients, potentially generating insensitivity toward women in childbirth [39, 48] through lack of attention to patient-provider dynamics, or even through direct rationalization of D&A [49]. Poor provider pay is thought to contribute to lack of RMC provision [17, 50, 51], as is lack of encouragement by facility leadership [17]. Provider demoralization and “moral distress” due to weak health systems, limited supplies, and understaffing have also been well described in relationship to lacking RMC [10, 17, 26, 49].

To date, there is no standardized or widely agreed upon way to define or measure either RMC or D&A. Scales for measurement of RMC have recently been proposed in Ethiopia [52] and in the USA and Canada [53], however, the tools are not yet validated in other contexts. Few studies have attempted to quantitatively identify patient and delivery factors associated with RMC. No identified studies have matched patient and provider interviews or any other form of modeling inclusive of linked patient and provider experience.

This novel study utilizes interviews linked between clients and providers from hospitals, health centers, and dispensaries to describe receipt and delivery of RMC, allowing for description of both patient and provider characteristics and their association with receipt of RMC. This study also contributes to the science around RMC by constructing measures of RMC based on domains from the WRA RMC Charter.

## Methods

### Study design and setting

We conducted cross-sectional surveys consisting of facility-based client exit interviews and provider interviews across 61 facilities (6 hospitals, 25 health centers, and 30 dispensaries) in Kigoma Region, Tanzania from April 30 to July 1, 2016.

Kigoma Region covers 45,066 km^2^ and is located in the northwest corner of Tanzania, bordering Lake Tanganyika, the Democratic Republic of Congo, and Burundi. Kigoma Region’s population in 2012 was 2,127,930 with an annual growth rate of 2.4% and 370,374 households [54]. Approximately 83% of the population live in rural areas where farming is the primary economic activity [54]. Nine out of 10 adults in Kigoma Region have attained a primary school education [54]. Less than two-thirds of births (62.8%) in Kigoma region occur in a health facility [55].

During our study, the Ministry of Health, Community Development, Gender, the Elderly and Children (MoHCDGEC) was implementing a number of efforts to improve maternal health in Tanzania. These efforts included the *National Roadmap Strategic Plan to Accelerate Reduction of Maternal, Newborn and Child Deaths in Tanzania 2008–2015*, the *Big Results Now* (BRN) initiative, and Wazazi Nipendeni (“Parents Love Me”; a safe motherhood multimedia campaign). Additionally, since 2006, the *Project to Reduce Maternal Deaths in Tanzania* has worked in Kigoma Region with the aim of decreasing maternal mortality.

### Sampling and data collection

#### Facility sampling

All hospitals (*n* = 6) and non-refugee camp health centers (*n* = 25) in Kigoma Region were included in the study. A sample of 30 dispensaries (of the approximately 163 dispensaries conducting deliveries in the region) was selected using the following criteria: 1) had an estimated 180 or more births per year; 2) had two or more onsite health providers; 3) was a site for BRN or project partner facility improvements, 4) referred patients to one of the 25 health centers; and 5) to maximize geographic distribution.

#### Provider sampling

The sampling frame for the provider survey comprised a list of all health care providers in the selected facilities. Providers were recruited if they were available during the study period and routinely provided labor and delivery care services. Providers were categorized into three cadres: 1) clinicians [Assistant Medical Officers/Clinical Officers/Assistant Clinical Officers], nurses/midwives [Nurse Officers/Assistant Nurse Officers/Registered Nurses/Midwives/Enrolled Nurses], other staff [Medical Attendants/Maternal and Child Health Aides]). Medical Doctors and specialists were excluded from participation due to the small number in the region. A sample of 189 provider interviews was needed to detect a 5% relative mean change in key variables of interest with 90% power and an *alpha* of 0.05.

#### Client sampling

Convenience sampling was used to enroll women as they exited delivery care services. Clients were eligible if they were 15 to 49 years of age and received delivery care services at the facility. Due to the focus of the project on routine labor and delivery care, clients were excluded if they delivered at home or on the way to the facility, had a cesarean section delivery, or experienced a stillbirth or neonatal death. A sample of 908 client interviews was needed to detect a 15% absolute difference in the variables of interest 90% power and an *alpha* of 0.05 (assuming a 50% reference proportion).

### Interview procedures and study tools

Interview guides were developed in English and translated into Swahili. Questionnaires were pre-tested in January 2016. Final questionnaires were translated from English to Swahili and back-translated to English. Informed consent was obtained from each respondent and confirmed with the respondent’s thumbprint. All client and provider interviews were administered face-to-face by an interviewer in Swahili. Interviews were conducted at the facility on the day of discharge, most commonly on the same day of delivery or the following day. The *Client Post-Delivery Exit Interview Questionnaire* captured sociodemographic characteristics, perceptions of and satisfaction with services, and pregnancy history and intention. The *Provider Interview Questionnaire* and *Self-administered Knowledge Test* were designed to capture information about provider demographic characteristics, education and training, supervision and mentorship, clinical knowledge, perceptions of the work environment, and current labor and delivery practices.

### Development of respectful maternity care measure

An initial 29 items were taken from the survey data to develop the RMC measure; these items were chosen based on domains from the WRA Respectful Maternity Care Charter and previously published research on RMC [11–14, 19–21, 33, 40]. The RMC items are described in detail in Table 1. Items representing disrespect (rather than respect) were reverse coded prior to inclusion. In initial scale reliability testing, no items were found to be redundant or negatively associated with the scale. Four items with low item-to-scale correlations were dropped (*Discharge time*, *Facility supplies, Facility cleanliness*, and *Wait time*). The remaining 25-item measure displayed strong internal consistency with Cronbach’s alpha of 0.83 and an inter-item correlation of 0.17. In support of criterion validity, Spearman testing found that the RMC measure was positively associated with the variable *Client satisfaction with care* (*rho* = 23.8, *p*-value < 0.001).

**Fig. 1:**
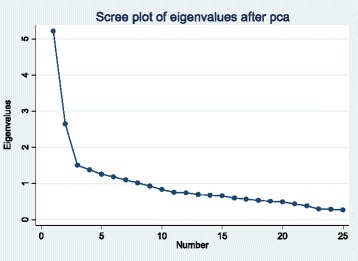
Scree plot of eigenvalues for Principal Components Analysis of Respectful Maternity Care survey items—Kigoma Region, Tanzania, April–July 2016

The 25 items were then entered into a Principal Components Analysis (PCA) to establish dimensionality of the scale with the aim of retaining the maximum amount of variance possible. The mean Kaiser-Meyer-Olkin measure of sampling adequacy was 0.81 and all individual item measures were greater than 0.68, indicating strong relationships among scale items [56]. Visualization of the scree plot supported a three-component solution for RMC (Fig. 1); client RMC scores were calculated for each of the first three components.

Items that loaded highest on the first principal component included *Advise on comfort measures*, *Friendliness*, *Visit regularly*, and *Pay close attention*. This first component was therefore termed the RMC Dimension 1 (RMC-D1), defined by the domains of Friendliness, Comfort, and Attention. Items that loaded highest on the second principal component included *Consent before procedures/exams*, *Explain what will happen*, *Explain procedures/exams beforehand*, and *Post-delivery counseling index*. This second component was therefore termed RMC Dimension 2 (RMC-D2), defined by the domains of Information and Consent. Items that loaded highest on the third principal component included *Absence of physical abuse*, *Absence of emotional abuse*, *Encouragement*, and *Kindness*. This component was therefore termed RMC Dimension 3 (RMC-D3), defined by Non-abuse and Kindness.

### Outcome variables

The outcome variables of interest included the continuous variables *RMC-D1 score*, *RMC-D2 score*, and *RMC-D3 score* to represent receipt of three dimensions of RMC.

### Independent variables

#### Client-level

The client-level variables of interest included:

- *Client age*: 15 to 19, 20 to 29, 30 to 39, 40 to 49 years of age, age unknown by client;
- *Literacy:* Able to read and write, Unable to both read and write;
- *Highest education attended*: No education, primary, secondary, college or university;
- *Total live births*: Two or fewer, three or more;
- *Marital status*: Not in union, in union;
- *Frequency of attendance at religious services*: Less than once a week, once a week or more often;
- *Companion in labor*: No, yes;
- *Companion at time of delivery*: No, yes;
- *Self-reported delivery complications*^1^: No, yes; and
- *SES*^2^: Low, low middle, middle, high middle, high wealth.

#### Provider-level

The provider-level variables of interest included:

- *Provider age*: 20 to 29, 30 to 39, 40 to 49, 50 years or older;
- *Sex*: Male, female;
- *Highest education completed*: Primary, secondary, college or university;
- *Cadre*: Clinicians, nurses/midwives, other staff;
- *Years in cadre*: Continuous;
- *Years at the facility*: Continuous;
- *Work hours per week*: Continuous;
- *Number of deliveries attended in last month*: One to 10, 11 to 20, 21 to 30, 31 or more, Don’t know;
- *Has on-site supervisor*: No, yes;
- *Job satisfaction*: Very satisfied, a little satisfied, neither satisfied nor dissatisfied, a little dissatisfied, very dissatisfied;
- *Perception paid fairly for job duties*: No, yes;
- *Perception of adequacy of training for job duties*: No, yes;
- *Perception in-service training has helped job performance*: No, yes;
- *Access to Electronic mentoring opportunities*: Access to zero, one, two, or three opportunities related to e-learning, emergency call system, and teleconference;
- *Recent complications summative index*: Has dealt with zero, one, two, three, or four types of complications in the last month related to hemorrhage, eclampsia, obstructed labor, and puerperal sepsis;
- *Delivery ever-training summative index*^3^*:* Pre- or in-service delivery training in 25 items, continuous;
- *Delivery pre-service summative index*^3^*:* Pre-service delivery training in 25 items, continuous;
- *Delivery in-service summative index*^3^*:* In-service delivery training in 25 items, continuous;
- *Recent delivery practice summative index*^*3*^*:* Provision of delivery services in 25 items in the last 3 months, continuous; and
- *Clinical knowledge test score*: Percent correct on 64 knowledge questions on the topics of antenatal care, routine delivery, newborn, complications, partograph, and postpartum.

### Analytic approach

Client and provider data were matched by asking providers on duty at the time of the delivery and by asking clients which provider most commonly provided their care; only matched client and provider interviews were included in the analysis. Data analyses were conducted using Stata 14.1. Bivariate analyses were conducted to identify client and provider variables associated with the outcome variables of interest; variables with a significant unadjusted relationship (*p* < 0.10) with the dependent variables were included in multivariate modeling. Multilevel, mixed-effects generalized linear models were fitted for the first three RMC PCA scores (*RMC-D1 score*, *RMC-D2 score*, and *RMC-D3 score*) to identify variables with a significant adjusted relationship (*p* < 0.05). Clustering of data by facility was further accounted for through inclusion of a facility identification cluster variable.

## Results

From April 30–July 1, 2016, a total of 960 delivery clients and 361 providers (Clinicians *n* = 72, Nurses/midwives *n* = 188, Other staff *n* = 98) were interviewed. Following exclusion of data from non-matched clients and providers, data from 935 delivery clients and 249 providers (Clinicians *n* = 69, Nurses/midwives *n* = 176, Other staff *n* = 85) were used in the analysis.

### Descriptive characteristics

Half of clients were 20 to 29 years of age (50.3%) and received care at a health center (50.6%). The majority of clients included in the study were married (91.0%), attended at least weekly religious services (86.4%), and have attended primary school education (67.3%). Nearly 45% of clients reported having a birth companion with them during labor (44.7%), while only 12% reported having a birth companion with them at the time of delivery. About 13% of clients reported that they experienced delivery complications (12.9%). (Table 2).

**Table 2:** Characteristics of delivery clients included in the respectful maternity care study sample—Kigoma Region, Tanzania, April–July 2016 (*n* = 935)

	Women, n (%)	95% CI
Age in years		
15 to19	163 (17.4)	15.1−20.0
20 to 29	470 (50.3)	47.1−53.5
30 to 39	251 (26.8)	24.1−29.8
40 to 49	38 (4.1)	3.0−5.5
Don’t know	13 (1.4)	0.8−2.4
Facility type		
Hospital	254 (27.2)	24.4−30.1
Health center	473 (50.6)	47.4−53.8
Dispensary	208 (22.3)	19.7−25.0
Marital status		
In a union	851 (91.0)	89.0−92.7
Not in a union	84 (9.0)	7.3−11.0
Frequency of attendance at religious services		
Attends at least weekly	808 (86.4)	84.1−88.5
Attends less often than weekly	127 (13.6)	11.5−15.9
Highest education attended		
No education	190 (20.3)	17.9−23.0
Primary	629 (67.3)	64.2−70.2
Secondary	100 (10.7)	8.9−12.8
College or University	16 (1.7)	1.0−2.8
Literacy		
Able to read and write	663 (70.9)	67.9−73.7
Unable to both read and write	264 (28.2)	25.4−31.2
Missing or refused	8 (0.9)	0.4−1.7
Socioeconomic status		
Low wealth	185 (19.8)	17.4−22.5
Low middle wealth	168 (18.0)	15.6−20.6
Middle wealth	186 (19.9)	17.5−22.6
High middle wealth	201 (21.5)	19.0−24.3
High wealth	195 (20.9)	18.4−23.6
Total live births		
Mean (SD)	3.3 (2.3)	3.1−3.4
Companion in labor		
Yes	418 (44.7)	41.5−47.9
No	517 (55.3)	52.1−58.5
Companion at time of delivery		
Yes	112 (12.0)	10.0−14.2
No	823 (88.0)	85.8−90.0
Self-reported delivery complications		
Yes	121 (12.9)	10.9−15.3
No	814 (87.1)	84.7−89.1

NOTE: *CI* Confidence Intervals, *SD* Standard Deviation

With respect to characteristics of RMC, nearly all clients reported that they were greeted respectfully upon admission (96.3%), while less than half reported that the provider introduced themselves (45.6%). Two-thirds of clients reported the provider explained what to expect in labor (63.0%). Regarding procedures and exams, most clients reported that the provider asked for consent (80.4%), explained the procedures and exams ahead of time (70.7%), and gave them the results (87.5%). One-third of clients reported the provider encouraged them to have a companion (32.7%). About three-quarters of clients reported feeling comfortable asking the provider questions (75.4%) and reported believing the information they gave to the provider would remain confidential (77.2%). Nearly all clients reported receiving privacy during exams and counseling (94.2%), although a few reported that other clients could overhear their conversations with the provider (7.9%). On average, clients received 6.7 out of 12 post-delivery counseling elements (Tables 1 and 3).

**Table 3:** Receipt of respectful care elements among clients included in the study sample—Kigoma Region, Tanzania, April–July 2016 (*n* = 935)

	Clients, n (%)	95% CI
Greeted respectfully		
Yes	900 (96.3)	94.8−97.3
No	35 (3.7)	2.7−5.2
Introduced themselves		
Yes	426 (45.6)	42.4−48.8
No	509 (54.4)	51.2−57.6
Explained what to expect		
Yes	589 (63.0)	59.8−66.0
No	346 (37.0)	34.0−40.2
Right amount of information given		
Yes	808 (86.4)	84.1−88.5
No	127 (13.6)	11.5−15.9
Consent before procedures/exams		
Yes	752 (80.4)	77.8−82.9
No	183 (19.6)	17.1−22.2
Explained procedures/exams beforehand		
Yes	661 (70.7)	67.7−73.5
No	274 (29.3)	26.5−32.3
Information given on results of procedures/exams		
Yes	818 (87.5)	85.2−89.5
No	117 (12.5)	10.5−14.8
Encouraged companion		
Yes	306 (32.7)	29.8−35.8
No	629 (67.3)	64.2−70.2
Client felt comfortable asking questions		
Yes	705 (75.4)	72.5−78.1
No	230 (24.6)	21.9−27.5
Client believes information will remain confidential		
Yes	722 (77.2)	74.4−79.8
No	213 (22.8)	20.2−25.6
Received privacy for exams and counseling		
Yes	881 (94.2)	92.5−95.6
No	54 (5.8)	4.4−7.5
Client believes other clients could hear conversations with provider		
Yes	74 (7.9)	6.3−9.8
No	861 (92.1)	90.2−93.7
Post-delivery counseling index, possible range 0 to 12		
Mean (SD)	6.7 (3.4)	6.4−6.9
Providers are friendly		
Yes	882 (94.3)	92.7−95.6
No	53 (5.7)	4.4−7.3
Perception of kindness		
Very kind	711 (76.0)	73.2−78.7
A little kind, Neither kind nor unkind, a little unkind, very unkind	224 (24.0)	21.3−26.8
Perception of encouragement		
Very encouraging	742 (79.4)	76.6−81.8
A little encouraging, Neither encouraging nor discouraging, a little discouraging, very discouraging	193 (20.6)	18.2−23.4
Provider advised client about comfort measures		
Yes	829 (88.7)	86.5−90.5
No	106 (11.3)	9.5−13.5
Provider Comfort Index, possible range 0 to 6		
Mean (SD)	1.3 (0.9)	1.3−1.4
Provider paid close attention during labor		
Yes	874 (93.5)	91.7−94.0
No	61 (6.5)	5.1−8.3
Provider visited regularly in labor		
Yes	865 (92.5)	90.6−94.0
No	70 (7.5)	6.0−9.4
Provider came when client called for them		
Yes	913 (97.7)	96.4−98.5
No	22 (2.4)	1.6−3.6
Physical abuse		
Yes	12 (1.3)	0.7−2.2
No	923 (98.7)	97.8−99.3
Emotional abuse		
Yes	25 (2.7)	1.8−3.9
No	910 (97.3)	96.1−98.2
Provider present at time of delivery		
Yes	916 (98.0)	96.8−98.7
No	19 (2.0)	1.3−3.2
Respectful maternity care dimension 1 score		
Low (below mean)	337 (36.0)	33.2−39.2
High (at or above mean)	598 (64.0)	60.8−67.0
Respectful maternity care dimension 2 score		
Low (below mean)	430 (46.0)	42.8−49.2
High (at or above mean)	505 (54.0)	50.8−57.2
Respectful maternity care dimension 3 score		
Low (below mean)	428 (45.8)	42.6−49.0
High (at or above mean)	507 (54.2)	51.0−57.4

NOTE: *CI* Confidence Intervals, *SD* Standard Deviation

Most clients reported that the provider was friendly (94.3%), and about three-quarters of clients reported the provider was very kind (76.0%) and very encouraging (79.4%). Nearly nine of 10 clients reported the provider advised them about comfort measures (88.7%); however, receipt of comfort measures from the provider was low at an average of less than two out of six comfort measures (1.3). Clients overwhelmingly reported the provider paid close attention to them during labor (93.5%) and came when they called for them (97.7%). Physical and emotional abuse was reported infrequently by clients at 1.3% and 2.7%, respectively. (Table 3).

Four in 10 providers were 20 to 29 years of age (41.0%), while one-fifth of providers were 50 years or older (21.7%). The majority of providers included in the study were female (64.7%), college/university educated (66.7%), and in the nurse/midwife cadre (61.0%). On average, providers reported working about 10.3 years in their cadre and 7.5 years at their facility, and reported working an average of 54.8 work hours per week. Two-thirds of providers (63.9%) reported conducting from one to 10 deliveries in the last month. Providers reported receiving in-service training on an average of 8 training elements; almost 9 of 10 providers reported in-service training has helped their job performance. Nearly half of providers reported not having access to electronic mentoring opportunities (48.2%). Less than half of providers stated they were satisfied with their job (44.6%), and less than one-fifth of providers feel they are paid fairly for their job duties (18.5%). On average, providers correctly answered 55.1% of the clinical knowledge questions. (Table 4).

**Table 4:** Characteristics of Providers included in the Respectful Maternity Care Study Sample—Kigoma Region, Tanzania, April–July 2016 (*n* = 249)

	Providers, n (%)	95% CI
Age in years		
20 to 29	102 (41.0)	34.8−47.1
30 to 39	37 (14.9)	10.4−19.3
40 to 49	56 (22.5)	17.3−27.7
50 or older	54 (21.7)	16.5−26.8
Sex		
Female	161 (64.7)	58.7−70.6
Male	88 (35.3)	29.4−41.3
Highest education completed		
Primary	12 (4.8)	2.1−7.5
Secondary	71 (28.5)	22.9−34.2
College/university	166 (66.7)	60.8−72.6
Cadre		
Clinician	34 (13.7)	9.4−17.9
Nurse/midwife	152 (61.0)	54.9−67.1
Other staff	63 (25.3)	19.9−30.7
Years in cadre		
Mean (SD)	10.3 (9.4)	9.2−11.5
Years at the facility		
Mean (SD)	7.5 (9.6)	6.3−8.7
Facility type		
Hospital	59 (23.7)	18.4−29.0
Health center	135 (54.2)	48.0−60.4
Dispensary	55 (22.1)	16.9−27.3
Work hours per week		
Mean (SD)	54.8 (14.6)	53.0−56.6
Number of deliveries attended in last month		
1 to 10	159 (63.9)	57.7−69.6
11 to 20	56 (22.5)	17.7−28.1
21 to 30	10 (4.0)	2.2−7.3
More than 30	11 (4.4)	2.5−7.8
Don’t know	13 (5.2)	3.0−8.8
Delivery ever-training summative index, possible range 0 to 25		
Mean (SD)	17.7 (5.02)	17.1−18.3
Delivery pre-service summative index, possible range 0 to 25		
Mean (SD)	15.6 (7.1)	14.7−16.4
Delivery in-service summative index, possible range 0 to 25		
Mean (SD)	8.4 (7.0)	7.6−9.3
Recent delivery practice summative index (in last 3 months), possible range 0 to 25		
Mean (SD)	14.9 (4.76)	14.3−15.5
Perception in-service training has helped job performance		
Yes	221 (88.8)	84.2−92.1
No	28 (11.2)	7.9−15.8
Recent complications summative index (in last 1 month)		
0 of 4 types of complications dealt with - postpartum hemorrhage, eclampsia, obstructed labor, puerperal sepsis (reference)	84 (33.7)	28.1−39.9
1 of 4	70 (28.1)	22.8−34.1
2 of 4	41 (16.5)	12.3−21.6
3 of 4	34 (13.7)	9.9−18.5
4 of 4	20 (8.0)	5.2−12.2
Access to electronic mentoring opportunities		
No access to any of 3 types of electronic mentoring – emergency call system, e-learning, teleconference	120 (48.2)	42.0−54.4
Access to 1 type	62 (24.9)	19.9−30.7
Access to 2 types	38 (15.3)	11.3−20.3
Access to 3 types	29 (11.7)	8.2−16.3
Job satisfaction		
Very satisfied	31 (12.5)	8.9−17.2
A little satisfied	80 (32.1)	26.6−38.2
Neither satisfied nor dissatisfied	31 (12.5)	8.9−17.2
A little dissatisfied	70 (28.1)	22.8−34.1
Very dissatisfied	37 (14.9)	10.9−19.9
Perception paid fairly for job duties		
Yes	46 (18.5)	14.1−23.8
Perception of adequacy of training for job duties		
Yes	169 (67.9)	61.8−73.4
No	80 (32.1)	26.6−38.2
Has an on-site supervisor		
Yes	172 (69.1)	63.0−74.5
No	77 (30.9)	24.5−37.0
Clinical knowledge test score, % correct		
Mean (SD)	55.1 (13.4)	53.4−56.8

NOTE: *CI* Confidence Intervals, *SD* Standard Deviation

### Receipt of respectful maternity care dimension 1 (RMC-D1): *Friendliness, Comfort, and Attention*

Results of bivariate analyses for RMC-D1 – *Friendliness, Comfort, and Attention*, are displayed in Appendix. Based on bivariate analyses for RMC-D1, the following variables were included in the multivariable model: *Client age, Total live births*, and *Self-reported delivery complications*; *Provider cadre*, *Ever-training summative index score*, *Delivery pre-service summative index score*, *Recent delivery practice summative index score*, *Number of deliveries attended in last month, Recent complications summative index score*, *Access to electronic mentoring opportunities, Perception paid fairly for job duties*, and *Perception in-service training has helped job performance*.

In multi-level, multivariate regression analyses, clients aged 30 to 39 years and clients aged 40 to 49 years had significantly higher RMC-D1 scores compared to clients 15 to 19 years (Coefficient [Coef] 0.63, 95% Confidence Intervals [CI] 0.14–1.13; Coef 0.79, 95% CI 0.18–1.39, respectively). Clients who reported that they experienced delivery complications had significantly lower RMC-D1 scores compared to clients who did not report complications (Coef -0.41, 95% CI -0.72-[-0.10]). The client variable of *Total live births* was not found to have a significant adjusted association with *RMC-D1 score*. (Table 5).

**Table 5:** Multilevel Mixed-Effects Generalized Linear Regression Analysis for Receipt of Respectful Care—Kigoma Region, Tanzania, April–July 2016 (Clients *n* = 935, Providers *n* = 249)

	Respectful Maternity Care Dimension 1: Friendliness, Comfort, and Attention		Respectful Maternity Care Dimension 2: Information and Consent		Respectful Maternity Care Dimension 3: Non-abuse and Kindness	
Fixed Effects - Client Level Variables	Adjusted Coefficient (95% CI)	*P*-value	Adjusted Coefficient (95% CI)	*P*-value	Adjusted Coefficient (95% CI)	*P*-value
Client age in years						
15 to 19 (reference)						
20 to 29	0.29 (−0.14−0.71)	0.185	−0.20 (−0.45−0.06)	0.131	0.01 (−0.27−0.29)	0.927
30 to 39	0.63 (0.14−1.13)	0.012	−0.17 (−0.52−0.19)	0.361	−0.02 (−0.30−0.27)	0.910
40 to 49	0.79 (0.18−1.39)	0.011	−0.22 (−0.90−0.45)	0.516	−0.20 (−0.67−0.27)	0.393
Don’t know	0.51 (−0.09−1.10)	0.094	0.29 (−0.12−0.70)	0.165	−0.85 (−1.87−0.17)	0.103
Highest education attended						
No education (reference)						
Primary	NA		−0.19 (−0.43−0.05)	0.128	NA	
Secondary	NA		0.20 (−0.27−0.68)	0.402	NA	
College or University	NA		−0.24 (−0.78−0.30)	0.385	NA	
Total live births						
0 to 2 (reference)						
3 or more	−0.14 (−0.45−0.17)	0.363	−0.13 (−0.39−0.14)	0.350	NA	
Socioeconomic status						
Low wealth (reference)						
Low middle wealth	NA		−0.12 (−0.47−0.22)	0.486	NA	
Middle wealth	NA		−0.02 (−0.31−0.26)	0.872	NA	
High middle wealth	NA		−0.24 (−0.51−0.04)	0.089	NA	
High wealth	NA		−0.12 (−0.49−0.26)	0.540	NA	
Frequency of attendance at religious services						
Attends less often than weekly (reference)						
Attends at least weekly	NA		−0.31 (−0.60−[−0.02])	0.035	NA	
Marital status						
Not in a union (reference)						
In a union	NA		NA		0.27 (−0.10−0.64)	0.159
Companionship in labor						
No (reference)						
Yes	NA		0.37 (0.06−0.68)	0.020	0.12 (−0.08−0.31)	0.253
Companionship at time of delivery						
No (reference)						
Yes	NA		0.10 (−0.32−0.52)	0.635	NA	
Self-reported delivery complications						
No (reference)						
Yes	−0.41 (−0.72−[−0.10])	0.010	NA		−0.29 (−0.60−0.02)	0.071
Fixed effects - Provider-level variables						
Provider age in years						
20−29 (reference)						
30−39	NA		−0.34 (−0.63−[−0.05])	0.023	0.14 (−0.06−0.34)	0.178
40−49	NA		−0.58 (−0.86−[−0.29])	0.000	0.03 (−0.20−0.27)	0.777
50+	NA		−0.09 (−0.41−0.23)	0.585	0.34 (0.09−0.58)	0.007
Cadre						
Clinician (reference)						
Nurse/midwife	−0.46 (−0.89−[−0.03])	0.038	NA		−0.02 (−0.27−0.24)	0.881
Other staff	0.33 (−0.27−0.92)	0.287	NA		−0.10 (−0.45−0.25)	0.560
Training and practice						
Delivery ever-training summative index, possible range 0 to 25	0.03 (−0.04−0.09)	0.402	NA		0.01 (−0.01−0.03)	0.356
Delivery pre-service summative index, possible range 0 to 25	0.01 (−0.04−0.06)	0.730	NA		NA	
Delivery in-service summative index, possible range 0 to 25	NA		NA		0.00 (−0.01−0.02)	0.550
Recent delivery practice summative index (in last 3 months), possible range 0 to 25	−0.02 (−0.07−0.02)	0.333	NA		NA	
Number of deliveries attended in last month						
1 to 10						
11 to 20	−0.35 (−0.67−[−0.02])	0.035	−0.30 (−0.61−0.01)	0.059	−0.02 (−0.22−0.19)	0.872
21 to 30	0.03 (−0.82−0.87)	0.947	−0.14 (−0.69−0.40)	0.599	−0.01 (−0.25−0.24)	0.953
More than 30	0.00 (−0.88−0.89)	0.994	0.40 (−0.30−1.11)	0.261	−0.43 (−0.89−0.03)	0.066
Don’t know	0.37 (−0.53−1.27)	0.420	−0.07 (−0.61−0.46)	0.792	−0.01 (−0.33−0.32)	0.971
Types of complications dealt with in last 1 month summative index						
0 of 4 types of complications dealt with - postpartum hemorrhage, eclampsia, obstructed labor, puerperal sepsis (reference)						
1 of 4	0.06 (−0.24−0.37)	0.692	NA		NA	
2 of 4	−0.57 (−1.28−0.15)	0.122	NA		NA	
3 of 4	−0.09 (−0.73−0.56)	0.794	NA		NA	
4 of 4	−0.67 (−1.55−0.22)	0.139	NA		NA	
Access to electronic mentoring opportunities						
Access to 0 of 3 types of electronic mentoring - emergency call system, e-learning, teleconference (reference)						
Access to 1 type	−0.08 (−0.64−0.48)	0.775	0.02 (−0.37−0.40)	0.938	0.10 (−0.10−0.29)	0.334
Access to 2 types	−0.17 (−0.85−0.51)	0.621	−0.05 (−0.48−0.38)	0.827	0.36 (0.07−0.65)	0.014
Access to 3 types	−0.74 (−1.49−0.01)	0.054	0.14 (−0.43−0.71)	0.631	0.25 (−0.08−0.59)	0.134
Work environment						
Perception paid fairly for job duties	0.46 (0.04−0.88)	0.032	0.37 (0.06−0.68)	0.019	NA	
Perception in-service training has helped job performance	−0.31 (−0.74−0.12)	0.156	NA		NA	
Work hours per week	NA		0.01 (0.00−0.02)	0.022	NA	
Random effects						
Provider-level variance (SE)	0.86 (0.29)		0.57 (0.11)		0.04 (0.04)	
Provider-level variance partition coefficient	0.18		0.24		0.03	
Level 1 units	935		935		935	
Level 2 units	249		249		249	
Log likelihood	−2032.0704		−1685.2573		−1492.6216	

NOTE: *CI* Confidence Intervals, *NA* Not Applicable, *SE* Standard Error

Clients of providers who perceived that they were paid fairly for their job duties had significantly higher RMC-D1 scores compared to clients of providers who perceived they are not paid fairly (Coef 0.46, 95% CI 0.04–0.88). Clients of Nurses/midwives had significantly lower RMC-D1 scores compared to clients of clinicians (Coef -0.46, 95% CI -089-[-0.03]). Clients of providers who reported attending 11 to 20 deliveries in the last month had significantly lower RMC-D1 scores compared to clients of providers who attended 1 to 10 deliveries (Coef -0.35, 95% CI -0.67-[-0.02]). Provider variables not found to have a significant adjusted association with *RMC-D1 score* included the following: *Delivery ever-training summative index*, *Delivery pre-service summative index*, *Recent delivery practice summative index, Recent complications summative index*, *Access to electronic mentoring opportunities*, and *Perception in-service training has helped job performance*. (Table 5).

The intraclass correlation (ICC) defines the proportion of the total variance that can be attributed to the hierarchal grouping by the provider variable. Net of all independent variables included in the final RMC-D1 model, 18% of the total variance (ICC = 0.18) is explained by the provider level.

### Receipt of respectful maternity care dimension 2 (RMC-D2): *Information and Consent*

Results of bivariate analyses for RMC-D2 – *Information and Consent*, are displayed in Appendix. Based on bivariate analyses for RMC-D2, the following variables were included in the multivariable model: *Client age*, *Highest education attended*, *Total live births*, *SES*, *Frequency of attendance at religious services*, *Companion in labor*, *Companion at time of delivery*; *Provider age*, *Number of deliveries attended in last month*, *Access to electronic mentoring opportunities*, *Perception paid fairly for job duties*, and *Work hours per week*.

In multi-level, multivariate regression analyses, clients who had a birth companion in labor had significantly higher RMC-D2 scores compared to clients who did not have a companion in labor (Coef 0.37, 95% CI 0.06–0.68). Clients who reported attending religious services at least weekly had significantly lower RMC-D2 scores compared to clients who reported less than weekly attendance (Coef -0.31, 95% CI -0.06-[-0.02]). Client-level variables found not to have a significant adjusted relationship with *RMC-D2 score* included *Age*, *Highest education attended*, *Wealth quintile*, *Total live births*, and *Companion at time of delivery*. (Table 5).

Clients of providers who perceived they were paid fairly for their job duties had significantly higher RMC-D2 scores compared to clients of providers who perceived they are not paid fairly (Coef 0.37, 95% CI 0.06–0.68). Clients of providers who reported working more hours per week had significantly higher RMC-D2 scores compared to clients of providers who work fewer hours (Coef 0.01, 95% CI 0.00–0.02). Clients of providers aged 30 to 39 and 40 to 49 years had significantly lower RMC-D2 scores compared to clients of providers aged 20 to 29 years (Coef -0.34, 95% CI -0.63-[-0.05]; Coef -0.58, 95% CI -0.86-[-0.29]). Provider variables not found to have a significant adjusted association with *RMC-D2 score* included *Number of deliveries attended in last month* and *Access to electronic mentoring opportunities*. Net of all independent variables included in the final RMC-D2 model, nearly one-quarter of the total variance (ICC = 0.24) is explained by the provider level. (Table 5).

### Receipt of respectful maternity care dimension 3 (RMC-D3): *Non-abuse and Kindness*

Results of bivariate analyses for RMC-D3 – *Non-abuse and Kindness*, are displayed in Appendix. Based on bivariate analyses for RMC-D3, the following variables were included in the multivariable model: *Client age*, *Marital status*, *Companion in labor, Self-reported delivery complications; Provider age, Cadre*, *Delivery ever-training summative index score*, *Delivery in-service summative index score*, *Number of deliveries attended in the last month*, and *Access to electronic mentoring opportunities*.

In multi-level, multivariate regression analyses, none of the client variables were found to have a significant adjusted association with *RMC-D3 score*. Clients of providers who were aged 50 years or more had significantly higher RMC-D3 scores compared to clients of providers in the 20 to 29 year age group (Coef 0.34, 95% CI 0.09–0.58). Clients of providers who reported access to two types of electronic mentoring had significantly higher RMC-D3 scores compared to clients of providers with no access to electronic mentoring opportunities (Coef 0.37, 95% CI 0.07–0.65). The provider variables of *Age*, *Cadre*, *Delivery ever-training summative index score, Delivery in-service summative index score*, and *Number of deliveries attended in the last month* were not found to have a significant adjusted association with *RMC-D3 score*. Net of all independent variables included in the final RMC-D3 model, only 3% of the total variance (ICC = 0.03) is explained by the provider level. (Table 5).

## Discussion

As maternal mortality and unskilled birth attendance continue to be high in sub-Saharan Africa, it is essential that the factors that influence health-seeking behavior and their determinants are better understood. In our study, we sought to identify the client and provider factors that predict receipt of three dimensions of RMC among delivery clients in Kigoma, Tanzania. The results provide insights into how dimensions of RMC, including receipt of friendly, comfort, and attention (RMC-D1), information and consent (RMC-D2), and non-abuse and kindness (RMC-D3) during labor and delivery are differentially influenced by characteristics of clients and their providers.

Client factors were significantly associated with the first two dimensions of RMC relating to friendliness, comfort, and attention (RMC-D1), and information and consent (RMC-D2). In our analyses, client age mattered; clients in their 30’s and 40’s perceived receiving significantly higher levels of RMC related to friendliness, comfort, and attention compared to clients in their teens. It is possible that health care providers interact and treat teens differently simply because they are younger than the providers are themselves, or it is possible that the providers perceive teens as *too young* to become a mother. Multiple qualitative studies and reviews thereof have pointed to younger women, particularly adolescents, as potential targets of discrimination and potential recipients of less respectful care [10, 11, 14]. Our findings are consistent with the analysis presented by Abuya et al. of D&A during childbirth in Kenya, where younger patients were significantly more likely to receive non-confidential care than older patients [13]. Our findings are in contrast to those by Kruk et al., however, who did not find age associated with receipt D&A in Tanzania [33].

Whether or not the client reported to have had delivery complications also influenced the first dimension of RCM; clients who reported delivery complications had a lower RMC score related to friendliness, comfort, and attention compared to those without perceived complications. There are two potential explanations for this finding: 1) the stress that providers experience during complications and emergencies may make them more likely to exhibit disrespectful behavior; or 2) the experience of complications lowers the client’s overall perception of the delivery experience.

Companionship in labor was found to be a positive factor for receipt of RMC related to information and consent; clients with a companion in labor received a higher level of RMC-D2. This finding is not surprising as providers may feel more accountable for providing better information and counseling when someone in addition to the client is present; a companion may also help increase the client’s understanding of information. Interestingly, more frequent attendance at religious services was a significant determinant of receipt of a lower level of RMC related to information and consent. It is possible that more religious women may interpret or receive these components differently than their less religious counterparts; alternatively, providers may display a particular bias against giving information to these women. Collectively, these findings provide a better understanding of how client characteristics—or provider perceptions and biases related to those characteristics—may influence provision of respectful care.

Provider factors were also significantly associated with the first two dimensions of RMC, and were the only factors of significance for the third non-abuse and kindness dimension of RMC. Nurses/midwives as compared to clinicians, and providers who attended 11 to 20 deliveries in the last month as compared to providers who attended fewer deliveries, were found to provide lower levels of RMC related to friendliness, comfort, and attention. These findings suggest that the high workload of labor and delivery care—commonly found among nurses/midwives—may lead to less positive interpersonal interactions with clients. Nurse/midwifes may experience prolonged contact with labor and delivery clients, as opposed to the often intermittent contact that clinicians experience, which may reduce a provider’s ability to give friendly, comforting, and attentive care day after day. Evidence bolstering this hypothesis comes from psychology research on the depleting effect of decision fatigue on subsequent self-control and active initiative [57]: providers may know that treating clients with respect is important and necessary, but may grow increasingly less able to provide respectful care with the demands of ongoing urgent clinical decision making without respite.

While job satisfaction was not found to be correlated with RMC, providers who perceived they were paid fairly for their work duties as compared to those who did not feel this way provided significantly better RMC related to friendliness, comfort, and attention, and RMC related to information and consent. These findings suggest that the perception of pay equity (versus pay *inequity*) positively influences interpersonal interactions and care provision, and likely reflects an underlying attitude that providers feel appreciated and motivated to do their work. Numerous qualitative studies and reviews thereof have highlighted health worker descriptions of low salaries as a particular stressful aspect of negative work environments resulting in unprofessional behavior [10, 35, 45, 58]. In Tanzania specifically, inadequate compensation for long hours, ineligibility for overtime pay, and lost opportunities to pursue other income-generating activities have been described as contributing to health care providers great dissatisfaction with their working environments [17].

Providers who reported working more as compared with fewer hours per week provided significantly higher levels of RMC related to information and consent. This finding may seem contradictory to the previously discussed finding that nurses/midwives and providers who conduct a higher number of deliveries provide less friendly, comforting, and attentive care. We contend, however, that friendliness/comfort/attention is a dissimilar construct from information sharing and consent, and therefore, it is not surprising to see disparate patterns of association. It is possible that providers who work more hours take more time to give information during labor and delivery care due to having more work hours, or they may have more opportunity from which to gain expertise communicating with and counseling clients through more frequent experience. Providers in their 30’s and 40’s provided lower levels of RMC related to information and consent, compared to providers in their 20’s. This finding suggests a possible shift in pre-service education whereby client counseling and consent have been emphasized in the education of more recent graduates or perhaps that younger providers simply have more motivation for sharing their knowledge with clients.

With respect to RMC related to non-abuse and kindness, the findings suggest that provider characteristics of age and access to electronic mentoring are protective. Providers aged 50 years and older provided higher levels of RMC care related to non-abuse and kindness than providers in their twenties. A potential explanation for this finding is that older providers, who are more experienced in labor and delivery, may be more patient and therefore less likely to respond negatively. Providers who reported access to two types of electronic mentoring, such as an emergency call line, teleconference, or e-learning, gave significantly better RMC related to non-abuse and kindness compared to clients of providers with no access to electronic mentoring opportunities. It is possible that providers with greater access are more likely to have received RMC training or that access to these types of mentorship opportunities improves provider’s underlying attitude toward work and the care of clients.

### Strengths and limitations

One strength of our study is the novel way in which we conceptualized and measured RMC. To-date, much of the research and literature around RMC has focused on D&A; we chose to focus on receipt of respectful care as our outcome of interest using the WRA RMC Charter as a conceptual framework. Using PCA to develop our outcomes allowed the identification of three dimensions of RMC in order to identify differences in determinants of RMC by broad dimension. In prior work, disrespect/respect has been operationalized in ways that limit interpretation and implications of findings. First, disrespect has been operationalized as a dichotomous variable where clients having experienced any one or more of a range of disrespectful practices are coded as a “1” [33]. Results from such an analysis are difficult to interpret because there is no differentiation in degree of disrespect; clients reporting not being greeted respectfully are considered to be equivalent in their experience of RMC with those who reported physical and emotional abuse by providers. Second, respect/disrespect has also been operationalized by running separate regression models for each item of respect/disrespect [13]. This approach results in generation of a large amount of data which may or may not be similar across models, making interpretation of findings and development of recommendations challenging.

A second strength of this study is the use of matched client and provider data. This was a particularly essential strategy given our findings that a larger number provider factors significantly influenced RMC compared to client factors. Identifying provider determinants of RMC allows for the development of recommendations aimed at specific provider characteristics (e.g., new graduates, cadre) or perceptions (e.g., age bias, religious bias) that would not be known otherwise. Another strength of this work is that we operationalized select independent variables in new ways. For example, provider-level *Delivery ever-training index*, *Delivery pre-service index*, and *Recent delivery practice index* variables were operationalized by summing multiple delivery care elements; these variables allowed the analyses to differentiate the importance of both dose and timing of training and practice to provision of RMC.

It is critical to understand the limitations of our work. First, the study was cross-sectional with non-random sampling, eliminating our ability to make causal inferences and generalize findings. Previously collected household-level Reproductive Health Survey (RHS) data in Kigoma from 2016 and a planned 2018 RHS in Kigoma provides a timely opportunity to analyze representative RMC data. Second, our questionnaire did not have items that fit into Article V and Article VII of the WRA RMC Charter, limiting our ability to account for certain known risk factors for receipt of non-respectful care. History of self-reported depression and history of past abuse or rape have both been associated with higher rates of abuse in health care settings in both high-resource [59, 60] and low-resource contexts [33]. Third, while using PCA as a means to create outcome variables has its strengths, it also has its limitations. Interpretation of coefficients is constrained; while we can easily interpret the direction and significance level of relationships, it is more problematic to understand to what extent a change in an independent variable has a *meaningful* change in receipt of RMC. Additionally, PCA rarely matches conceptual frameworks perfectly, especially for complex constructs such as RMC. Though strong patterns emerged in our PCA, not every item clustered with the most logical component (e.g., *Inform about findings of exam/procedures* loaded highest on the first component, not the second component, as expected).

The site of client interviews at facilities poses an additional important limitation, likely generating an underestimation of true prevalence of D&A and an overestimation of receipt of RMC. Two independent studies of D&A treatment during facility delivery in Tanzania have demonstrated markedly lower reports of D&A from interviewed clients when interviewed at the site of the facility, with significant increases in reporting upon follow up in the community [31, 33]. Another limitation of our data is that some women, particularly those who delivered in hospitals and health centers, may have had more than one care provider. We attempted to control for this issue by matching women with the provider who they reported spending the greatest amount of time with them. Due to budget and time constraints, our questionnaire items used in the development of RMC measures were not developed on the basis of formative work in Kigoma Region. Rather, these items were a compilation of commonly used RMC elements in prior research. Qualitative work ahead of this study may have uncovered local conceptualizations of RMC that would have been important to include in our measure. Finally, the provider-level variables only accounted for 3% of the variance of RMC-D3, and client variables did not have a significant adjusted relationship with this outcome; this suggests that the RMC-D3 model had a poor overall fit. It is important for future work to attempt to explore client and provider characteristics not considered in this study and potential reframing of the non-abuse and kindness dimension.

### Programmatic, policy, and research implications

These findings highlight potential areas of focus for programmatic and policy work, as well as future directions to move the RMC research agenda forward. Given our results that Nurses/midwives and providers who conduct a higher number of deliveries provide lower levels of RMC, improving the work environment for labor and delivery providers may improve delivery care. Strategies that aim to reduce workplace stress—including reduction of moral distress and decision fatigue—and improve providers’ perceptions of workplace support, self-efficacy in providing quality care, and underlying attitude toward work, may contribute to improved interpersonal interactions between clients and providers. Such approaches might include offering high frequency rotational schedules to give labor and delivery providers short-term respite away from providing maternity care. Strategies that increase access to mentoring and peer-to-peer learning opportunities (with fair access across cadres) may improve workplace support, self-efficacy, and enhance feelings of being a respected and valued member of the team. Pre-service and in-service training on RMC, as well as close mentorship following training, is essential to determine the influence of training on knowledge transfer and behavior change. Additionally, ensuring providers receive equitable pay, on-time, every time may increase provider’s sense of worth and underlying attitude towards work.

To move the RMC scientific agenda forward, additional research using matched patient and provider data may improve understanding of the relative importance of patient and provider determinants of RMC. In addition, studies embedded in conceptual models of RMC are needed that aim to standardize and validate measures of RMC. Measures that can be validated across cultural and geographic settings would be particularly valuable so that RMC data can be compared and synthesized across studies. Future analyses would be strengthened through the addition of interaction terms to illuminate the complexities of patient and provider relationships and how these influence respectful care. Given our hypothesis that moral distress and decision fatigue contribute to lack of RMC, future analyses would greatly benefit from inclusion of measures for these constructs. Furthermore, future research may benefit from over-sampling of clinician providers in order to increase the power to detect differences between clinicians and other cadres, and potential differences by gender that were not detected here. Finally, women in low-resource settings may have relatively low expectations of maternity care compared to women in middle- or high-resource settings. Measuring expectations of care and the influence of cultural and gender norms in future research would help advance our understanding of women’s experience of RMC and how expectations and context influence measurement and comparison of RMC across settings.

## Conclusion

Despite disrespectful maternity care being increasingly recognized as a key deterrent to women seeking facility-based deliveries, there is less consensus about the comparative importance of patient and provider determinants for RMC. Our findings demonstrate that patient and provider factors differentially influence three dimensions of RMC. Future research is needed that aims to standardize RMC measurement through the lens of a conceptual model of RMC and rooted in a human rights perspective. Strategies that promote more equitable pay, offer rotational schedules with short-term respite away from providing maternity care, and increased access to mentoring and peer-to-peer learning platforms may improve RMC and uptake of facility delivery in low-resource settings. An enhanced understanding of the relationships between patient and provider characteristics may improve the provision of quality labor and delivery services and should be considered in the design of maternity care programs, policies, and future research.

## Appendix

**Table 6:** Bivariate Associations between Client and Provider Characteristics and Receipt of Respectful Maternity Care—Kigoma Region, Tanzania, April–July 2016 (Clients n = 935, Providers *n* = 249)

	Respectful Maternity Care Dimension 1 – Friendliness, Comfort, and Attention		Respectful Maternity Care Dimension 2 – Information and Consent		Respectful Maternity Care Dimension 3 – Non-abuse and Kindness	
	Unadjusted Coefficient (95% CI)	*P*-value	Unadjusted Coefficient (95% CI)	*P*-value	Unadjusted Coefficient (95% CI)	*P*-value
Client level variables						
Age in years						
15 to 19 (reference)						
20 to 29	0.28 (−0.09−0.66)	0.142	−0.24 (−0.50−0.02)	0.074	0.05 (0.17−0.27)	0.652
30 to 39	0.55 (0.13−0.97)	0.010	−0.27 (−0.56−0.02)	0.072	0.05 (−0.19−0.29)	0.688
40 to 49	0.62 (−0.13−1.37)	0.105	−0.36 (−0.88−0.17)	0.181	−0.11 (−0.54−0.32)	0.616
Don’t know	0.22 (−0.99−0.42)	0.727	0.33 (−0.51−1.17)	0.446	−0.83 (−1.52−-0.14)	0.019
Highest education attended						
No education (reference)						
Primary	−0.06 (−0.41−0.30)	0.759	−0.31 (−0.56−[−0.07])	0.012	0.00 (−0.20−0.20)	0.997
Secondary	−0.11 (−0.64−0.42)	0.691	0.05 (−0.31−0.42)	0.769	−0.12 (−0.42−0.18)	0.426
College or University	0.44 (−0.66−1.53)	0.434	−0.34 (−1.09−0.40)	0.366	−0.35 (−0.97−0.27)	0.271
Literacy						
Cannot read or write, or can read or write, but not both (reference)						
Can read and write	0.23 (−0.07−0.54)	0.134	−0.15 (−0.37−0.06)	0.158	0.09 (−0.09−0.26)	0.333
Socioeconomic status						
Low wealth (reference)						
Low middle wealth	−0.37 (−0.82−0.07)	0.101	−0.17 (−0.48−0.14)	0.283	−0.06 (−0.31−0.20)	0.653
Middle wealth	−0.28 (−0.72−0.16)	0.212	−0.16 (−0.46−0.15)	0.317	−0.09 (−0.34−0.16)	0.492
High middle wealth	−0.25 (−0.69−0.18)	0.258	−0.34 (−0.64−[−0.04])	0.029	0.09 (−0.16−0.33)	0.485
High wealth	−0.01 (−0.48−0.45)	0.954	−0.17 (−0.49−0.16)	0.312	0.03 (−0.22−0.28)	0.814
Total live births						
0 to 2 (reference)						
3 or more	0.24 (−0.03−0.52)	0.082	−0.16 (−0.35−0.03)	0.097	0.06 (−0.10−0.21)	0.484
Marital status						
Not in a union (reference)						
In a union	0.19 (−0.30−0.67)	0.448	−0.28 (−0.61−0.06)	0.106	0.24 (−0.03−0.52)	0.085
Frequency of attendance at religious services						
Attends less often than weekly (reference)						
Attends at least weekly	0.28 (−0.12−0.69)	0.165	−0.39 (−0.67−0.12)	0.005	−0.07 (−0.30−0.16)	0.572
Companion in labor						
No (reference)						
Yes	0.15 (−0.17−0.46)	0.360	0.45 (0.23−0.66)	0.000	0.15 (−0.01−0.32)	0.065
Companion at time of delivery						
No (reference)						
Yes	−0.10 (−0.56−0.36)	0.675	0.31 (−0.01−0.63)	0.058	−0.12 (−0.37−0.13)	0.346
Self-reported delivery complications						
No (reference)						
Yes	−0.53 (−0.95−[−0.12])	0.012	−0.11 (−0.41−0.18)	0.439	−0.29 (−0.53−[−0.05])	0.016
Provider-level variables						
Age in years						
20 to 29 (reference)						
30 to 39	−0.05 (−0.62−0.52)	0.868	−0.43 (−0.85−0.02)	0.041	0.17 (−0.07−0.41)	0.175
40 to 49	−0.10 (−0.61−0.40)	0.687	−0.62 (−0.99−[−0.26])	0.001	0.13 (−0.08−0.35)	0.233
50 or older	0.23 (−0.30−0.76)	0.393	−0.07 (−0.46−0.31)	0.700	0.40 (0.17−0.63)	0.001
Sex						
Male (reference)						
Female	−0.31 (−0.72−0.10)	0.142	−0.21 (−0.51−0.10)	0.183	−0.09 (−0.27−0.09)	0.335
Highest education completed						
Primary (reference)						
Secondary	−0.64 (−1.65−0.36)	0.211	0.03 (−0.71−0.77)	0.942	0.31 (−0.15−0.76)	0.185
College/university	−0.49 (−1.46−0.48)	0.326	0.29 (−0.42−1.00)	0.426	0.15 (−0.29−0.59)	0.490
Cadre						
Clinician (reference)						
Nurse/midwife	−0.52 (−1.11−0.08)	0.087	−0.15 (−0.60−0.29)	0.501	−0.24 (−0.51−0.04)	0.091
Other staff	0.36 (−0.31−1.02)	0.290	0.13 (−0.37−0.64)	0.600	−0.29 (−0.60−0.02)	0.064
Years in cadre	0.01 (−0.01−0.03)	0.360	−0.01 (−0.02−0.01)	0.245	0.01 (−0.00−0.02)	0.119
Years at the facility	0.01 (−0.01−0.03)	0.200	−0.00 (−0.02−0.01)	0.824	0.01 (−0.00−0.02)	0.105
Work hours per week	0.01 (−0.00−0.02)	0.140	0.02 (0.01−0.03)	0.000	−0.00 (−0.01−0.00)	0.114
Delivery ever-training summative index, possible range 0 to 25	−0.04 (−0.08−[−0.01])	0.025	−0.00 (−0.03−0.03)	0.998	0.01 (−0.00−0.03)	0.097
Delivery pre-service summative index, possible range 0 to 25	−0.03 (−0.06−0.00)	0.034	0.00 (−0.02−0.02)	0.650	0.00 (−0.01−0.01)	0.624
Delivery in-service summative index, possible range 0 to 25	−0.01 (−0.04−0.01)	0.352	−0.01 (−0.03−0.01)	0.302	0.01 (0.00−0.02)	0.046
Recent delivery practice summative index, (in last 3 months), possible range 0 to 25	−0.07(−0.11−[−0.03])	0.000	0.00 (−0.03−0.03)	0.831	0.01 (−0.00−0.03)	0.124
Number of deliveries attended in last month						
1 to 10 (reference)						
11 to 20	−0.65 (−1.11−[−0.20])	0.005	−0.26 (−0.61−0.08)	0.129	0.03 (−0.17−0.23)	0.794
21 to 30	−0.34 (−1.26−0.57)	0.462	−0.06 (−0.75−0.63)	0.861	−0.05 (−0.45−0.35)	0.821
More than 30	−0.82 (−1.71−0.08)	0.074	0.60 (−0.07−1.27)	0.080	−0.37 (−0.76−0.03)	0.066
Don’t know	−0.10 (−0.93−0.73)	0.814	−0.20 (−0.82−0.43)	0.535	0.06 (−0.31−0.42)	0.766
Recent complications summative index (in Last 1 Month)						
0 of 4 types of complications dealt with: postpartum hemorrhage, eclampsia, obstructed labor, puerperal sepsis (reference)						
1 of 4	−0.21 (−0.71−0.28)	0.402	−0.06 (−0.44−0.32)	0.750	0.02 (−0.21−0.25)	0.874
2 of 4	−0.89 (−1.44−[−0.35])	0.001	−0.32 (−0.74−0.10)	0.134	−0.17 (−0.41−0.08)	0.186
3 of 4	−0.68 (−1.28−[−0.09])	0.025	−0.18 (−0.64−0.28)	0.438	−0.02 (−0.29−0.25)	0.858
4 of 4	−1.06 (−1.75−[−0.36])	0.003	−0.39 (−0.92−0.15)	0.159	0.18 (−0.13−0.48)	0.260
Access to electronic mentoring opportunities						
Access to 0 of 3 types of electronic mentoring: emergency call system, e-learning, teleconference (reference)						
Access to 1 type	−0.10 (−0.57−0.36)	0.664	−0.00 (−0.35−0.35)	0.994	0.13 (−0.07−0.34)	0.198
Access to 2 types	−0.35 (−0.92−0.22)	0.227	−0.03 (−0.45−0.40)	0.903	0.37 (0.12−0.62)	0.004
Access to 3 types	−0.92 (−1.53− −0.30)	0.003	0.43 (−0.03−0.89)	0.068	0.22 (−0.05−0.49)	0.111
Perception in-service training has helped job performance						
No (reference)						
Yes	−0.69 (−1.32−[−0.06])	0.033	−0.34 (−0.81−0.13)	0.157	0.13 (−0.16−0.42)	0.384
Job satisfaction						
Very dissatisfied (reference)						
A little dissatisfied	−0.07 (−0.68−0.54)	0.820	−0.03 (−0.49−0.42)	0.895	−0.05 (−0.32−0.22)	0.707
Neither satisfied nor dissatisfied	−0.16 (−0.93−0.61)	0.682	−0.40 (−0.97−0.17)	0.165	0.02 (−0.33−0.37)	0.916
A little satisfied	−0.45 (−1.06−0.15)	0.142	−0.22 (−0.67−0.23)	0.334	0.06 (−0.21−0.32)	0.680
Very satisfied	−0.09 (−0.83−0.65)	0.814	0.10 (−0.45−0.65)	0.730	0.15 (−0.18−0.48)	0.379
Perception paid fairly for job duties						
No (reference)						
Yes	0.57 (0.07−1.07)	0.027	0.46 (0.08−0.83)	0.016	−0.06 (−0.29−0.16)	0.584
Perception of adequacy of training for job duties						
No (reference)						
Yes	−0.02 (−0.44−0.40)	0.925	−0.16 (−0.47−0.15)	0.310	0.02 (−0.16−0.21)	0.810
Has an on-site supervisor						
No (reference)						
Yes	0.07 (−0.35−0.49)	0.732	−0.17 (−0.48−0.14)	0.292	−0.05 (−0.24−0.14)	0.599
Clinical knowledge test score	−0.63 (−2.06−0.81)	0.392	−0.31 (−1.38−0.76)	0.568	0.41 (−0.23−1.05)	0.211

NOTE: *CI* Confidence Intervals

## Abbreviations

BRN: Big results now
CDC: Centers for disease control and prevention
CI: Confidence intervals
Coef: Coefficient
D&A: Disrespect and abuse
ICC: Intraclass correlation
MOHCDGEC: Ministry of health, community development, gender, the elderly, and children
NBS: National bureau of statistics
PCA: Principal components analysis
RHS: Reproductive health survey
RMC: Respectful maternity care
RMC-D1: Respectful maternity care-dimension 1
RMC-D2: Respectful maternity care-dimension 2
RMC-D3: Respectful maternity care-dimension 3
SD: Standard deviation
SE: Standard error
USAID: United States agency for international development
WHO: World Health Organization
WRA: White ribbon alliance

## Footnotes

1 Women were asked if they had any complications during labor and delivery. The most common self-reported complications included postpartum hemorrhage, prolonged labor, retained placenta, malpresentation, and lacerations.

2 The variable for *SES* was developed using principal components analysis (PCA); household assets and characteristics were weighted based on their contribution to the first principal component and summed to create an index score representing five levels of relative household wealth [61].

3 Providers were asked, “Have you received pre-service training in […]?”; “Have you received in-service training in […]?”; and “Have you conducted […] in the last 3 months?” for the following 25 items: 1) Focused antenatal care; 2) Routine labor and delivery care; 3) Use the partograph; 4) Active management of the third stage of labor; 5) Manual removal of the placenta; 6) Beginning intravenous fluids; 7) Checking for anemia; 8) Administering intramuscular or intravenous magnesium sulfate for the treatment of server pre-eclampsia or eclampsia; 9) Administering intravenous antibiotics; 10) Administering misoprostol or other uterotonic; 11) Bimanual uterine compression (external); 12) Bimanual uterine compression (internal); 13) Suturing an episiotomy; 14) Suturing vaginal lacerations; 15) Suturing cervical lacerations; 16) Vacuum extractor; 17) Forceps; 18) C-section; 19) A blood transfusion; 20) Adult resuscitation; 21) Resuscitating a newborn with bag and mask; 22) Basic Emergency Obstetric and Neonatal Care (BEmONC); 23) Advanced Emergency Obstetric and Neonatal Care; 24) Administering antiretrovirals (ART) for Prevention of Mother-to-Child Transmission (PMTCT); and 25) Rapid diagnostic testing for HIV. Responses were summed to create four indices.
